# Temperature-Related Properties of Solid Birch Wood under Quasi-Static and Dynamic Bending

**DOI:** 10.3390/ma13235518

**Published:** 2020-12-03

**Authors:** Georg Baumann, Reinhard Brandner, Ulrich Müller, Cedou Kumpenza, Alexander Stadlmann, Florian Feist

**Affiliations:** 1 Vehicle Safety Institute, Graz University of Technology, 8010 Graz, Austria; florian.feist@tugraz.at; 2 Institute of Timber Engineering and Wood Technology, Graz University of Technology, 8010 Graz, Austria; reinhard.brandner@tugraz.at; 3 Institute of Wood Technology and Renewable Materials, Department of Material Sciences and Process Engineering, University of Natural Resources and Life Sciences Vienna, Austria (BOKU), 3430 Tulln a. d. Donau, Austria; ulrich.mueller@boku.ac.at (U.M.); cedou.kumpenza@boku.ac.at (C.K.); alexander.stadlmann@boku.ac.at (A.S.)

**Keywords:** birch, energy absorption, loading velocity, temperature-effects

## Abstract

The project WoodC.A.R. investigates the capabilities of wood and engineered wood-products (EWPs) for their application as a load-bearing material in automotive applications. For crash-relevant components, materials have to provide a high impact bending energy over a wide range of climatic conditions. This study investigates the effect of temperature on the bending behavior of solid birch wood beams (800 × 90 × 43 mm^3^) under quasi-static and dynamic loading. Specimens were exposed to a three-point bending test with lateral confinement, replicating the hypothetical installation environment in a car, at five temperature levels: −30 °C, 0 °C, +30 °C, +60 °C, and +90 °C. A cylindrical impactor (D = 254 mm, m = 91 kg) was propelled against the center of the beam with an initial velocity of 8.89 m/s (dynamic) and at a constant velocity of 10 mm/min (quasi-static), respectively. Specimens were conditioned in a freezer and a climate chamber, respectively. Temperature was monitored prior and during testing. Bulk density and global fiber deviation were determined afterwards. In both, the dynamic and the quasi-static load case maximum force slightly decreased with increasing temperature, but remained almost constant at temperatures exceeding +30 °C. On average, the maximum dynamic peak force level was twice as high as in quasi-static tests. In the quasi-static tests, the energy absorption remained constant at elevated temperatures (+30 °C to +90 °C) but decreased by about 50% at lower temperatures −30 °C and 0 °C. In the dynamic tests, the energy absorption remained almost constant throughout the entire temperature range.

## 1. Introduction

In order to make wood suitable for the purposes of mechanical and, in particular, for automotive engineering, it is important to understand how the material reacts under dynamic load or impact (see Müller [1] and Müller et al. [2]). Wood as a natural construction material has been mainly used and studied in the field of civil engineering in the last decades. Therefore, there is only limited data available with respect to impact loading. This is because loads in civil engineering applications are commonly considered to be quasi-static even if they are cyclic, e.g., for earthquakes, or dynamic, e.g., on bridge piers. Special design rules like the ÖNORM EN 1991-1-7–2014-09 [3], the ÖNORM EN 1991-2–2012-03 [4], and the ÖNORM EN 1991-3–2013-12 [5], which are regulating impact and cyclic load cases, refer to equivalent quasi-static loads.

**Figure 1:**
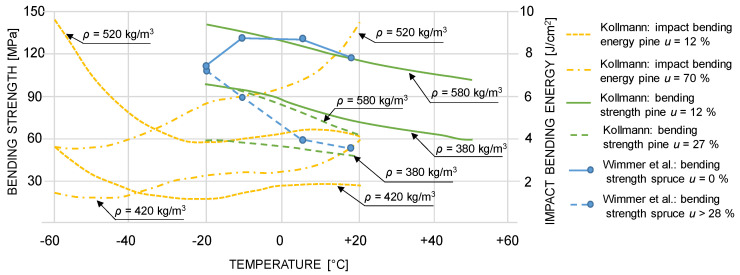
Overview of the mean bending strength and mean impact bending energy values of spruce and pine vs. temperature and at varying moisture contents and densities.

Studies on wooden structures under impact loading are scarce. Goubel et al. [6] and Murray [7] studied wooden guard rails and posts from yellow pine under impact loading. Shenoy et al. [8], O’Brien [9], Ruggiero et al. [10], Drane et al. [11], Manin et al. [12], Ruggiero et al. [13] investigated sports equipment under impact bending load, also considering the effects of moisture content (u) and temperature. Goubel et al. [6] conducted three-point bending impact tests on guard rails with a diameter of 200 mm at impact velocities between 1.39 m/s and 5.56 m/s. They showed that the variation of the moisture content shows a positive correlation with the variation of the peak acceleration. Wimmer et al. [14] focused on the quasi-static bending properties of small clear wood specimens made of Norway spruce (*Picea abies*) conditioned to temperatures in the range of −20 °C to +18 °C. Their tests also covered extreme cases of moisture content (u), namely oven-dry (*u* = 0%) and fully saturated state (all lumen filled with water; *u* >>> 100%). At a temperature of −20 °C, both the oven-dry and the saturated samples showed almost identical bending strength and stiffness. With increasing temperature, the strength and stiffness values of the saturated samples decreased almost linearly (55% and 25% loss in terms of bending strength and bending stiffness at +18 °C, respectively). For the oven-dry samples, the situation was found quite different: bending strength values at +18 °C and at −20 °C were identical, but slightly smaller than at temperatures in-between (−10 °C and +6 °C). In principle, the same applies to bending stiffness, although it is slightly lower at +18 °C than at −20 °C. Kollmann [15] provides values for the temperature- and moisture-related behavior of pine under quasi-static bending and impact bending. In the case of the quasi-static bending strength, the samples were tested at moisture contents of 12% and 27% and at temperatures between −20 °C and +50 °C. It is shown that the bending strength is significantly decreasing with increasing temperature. The effect is getting stronger with increasing moisture content. The samples with 12% moisture content show an average bending strength reduction of about 0.5%/°C while the bending strength of the samples with a moisture content of 27% is reduced by roughly 0.75%/°C. The impact bending energy characteristics were observed at temperatures between −60 °C and +20 °C and at moisture levels of 12% and 70%. The samples, which were conditioned at 12% moisture content, showed the highest impact bending energy values at −60 °C, and a significant decrease down to the lowest values at around −20 °C. At that point, the impact bending energy was slightly increasing again up to a temperature between +10 °C and +20 °C. In the case of the samples conditioned at 70% moisture content, the strength values were lowest at temperatures between −60 °C to −40 °C and highest at +20 °C. An overview of the bending strength and impact bending energy values from Wimmer et al. [14] and Kollmann [15] is provided in Figure 1.

Kretschmann and Green [16] investigated the effects of moisture content on the tensile, compression, bending, shear, and elastic properties as well as on the fracture toughness in Mode I and II on southern pine. According to their findings, the tensile strength parallel and perpendicular to the fiber as well as the fracture toughness in Mode I and Mode II are increasing with a decreasing moisture content showing a peak between about 7% to 13% moisture content. In the case of compression strength parallel and perpendicular to the fiber direction, the bending strength, the shear strength in fiber direction, and the elastic properties, the values are increasing with decreasing moisture content and are highest at roughly 4% moisture content.

Bucar and Merhar [17] conducted dynamic impact bending energy tests on Norway spruce (dimensions: 300 × 10 × 10 mm^3^), measuring both the energy absorption as well as the pendulum deceleration. According to their study, both the energy absorption and the integral of the acceleration signal show a good correlation (coefficient of determination R^2^ = 0.88). The correlation between the specimen’s density and the impact bending energy was low (linear regression model with a coefficient of determination R^2^ = 0.26). Furthermore, the correlation between the dynamic bending strength and the impact bending energy was investigated. Applying a linear regression model to fit the data obtained from tests showed a coefficient of determination of R^2^ = 0.51.

The majority of studies investigated coniferous wood species like pine or spruce. Data on hardwood, like birch, is scarce: Tukiainen and Hughes [18] compared the fracture behavior of Norway spruce and birch (Betula pendula) in Mode I using compact tension samples. The samples were conditioned at +22 °C and +50 °C with moisture contents of 14% and at completely saturated states. The highest fracture mechanical properties were obtained at +22 °C and at a moisture content of 14%. The samples conditioned at +50 °C and at complete fiber saturation showed the lowest values. Based on their results, it can be concluded that birch wood in terms of fracture mechanical properties, except for the specific fracture energy, is more sensitive to changes in temperature and moisture content than spruce.

Gerhards [19] has presented an overview study on the effects of moisture content and temperature and how they are related to the mechanical properties. In his study, he described average effects and trends resulting from observations of many different wood species. He pointed out that the compressive strength parallel to fiber is between 2.5 and 4.5 times more sensitive to changes in moisture content than the tensile strength parallel to fiber.

To sum up, in the past, several studies on the mechanical properties of wood affected by different settings of temperature and moisture were conducted. However, these studies mainly focused on coniferous wood species and specimens on a small clear wood scale. For this study, birch wood was chosen due to its favorable elastic and strength properties (see Heräjärvi [20]). Birch is a wood species which also saw extensive use as a lightweight construction material in historic aircrafts. In this field of application, it was often used in combination with other wood species like in the De Havilland Mosquito with outer birch layers and an inner balsa core (see Falconer and Rivas [21]. Therefore, birch is also considered as well suited for the task of developing bio-based structural components in vehicles. Furthermore, there is also a wide availability of this wood species, especially in Scandinavia. Due to the diffuse porous nature of birch, it is also suitable for the production of rotatory cut veneers, which are the basis for products like LVL (laminated veneer lumber) or plywood. In order to use hardwoods like birch for structural, load-bearing components in the automotive sector, it is necessary to investigate several mechanical and climate-related aspects. Therefore, impact tests under boundary conditions which are relevant in the automotive industry in terms of dimensions and temperature levels, were conducted. The aim of the present study is to validate if properties of birch wood at component size exposed to quasi-static and dynamic bending are similar to the properties of small birch clear wood specimens as found in previous tests. Furthermore, the temperature effects on the bending properties and in particular on the energy-absorbing capability were investigated on a mean value level.

## 2. Materials and Methods

### 2.1. Wood Specie, Quality, and Test Matrix

**Figure 2:**
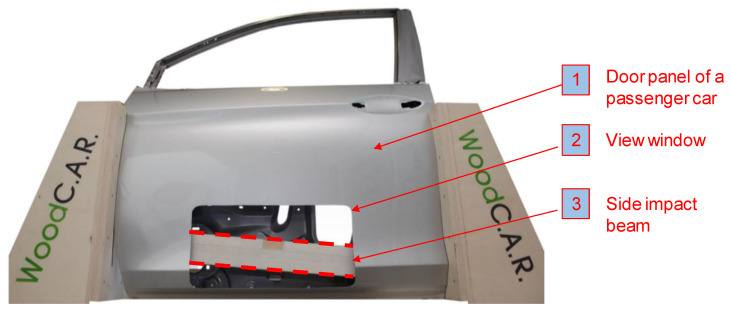
Demonstrator of a passenger car door panel with an integrated side-impact beam.

For the preparation of the samples, flat and half rift-sawn planks of visually graded, air-dried birch (*Betula pendula* sourced from J. u. A. Frischeis GmbH, Stockerau, Austria) were used by applying strict grading rules according to ISO 3129–2019 11 [22]. Therefore, growth features like knots or significant fiber deviations as well as drying cracks were avoided, which means that the material fulfilled almost clear wood standards. However, no clear distinction between Radial-Longitudinal and Tangential-Longitudinal direction was taken into account. In most sources from literature like Wagenführ [23], Grabner [24], Sell [25], and Kretschmann [26], no distinction is made between the two material directions with respect to bending strength and impact bending energy. This is due to the negligible differences between these two directions. The samples were sawn to dimensions (800 × 90 × 43 mm^3^) (length × width × thickness) so that the global fiber was nominally running parallel to the length of the planks. The chosen dimensions resulted from the installation space available for a side impact beam inside a car door. A side impact beam (shown in Figure 2) is a crash-absorbing component installed in a car door which protects the occupants in case of a side-impact collision (see Shaharuzaman [27] and Baumann et al. [28]).

Six replicates were tested at five temperature levels, ranging from nominally −30 °C to +90 °C, under both quasi-static and dynamic conditions [29,30]. An overview of the test matrix is given in Table 1.

### 2.2. Conditioning of the Samples

**Figure 3:**
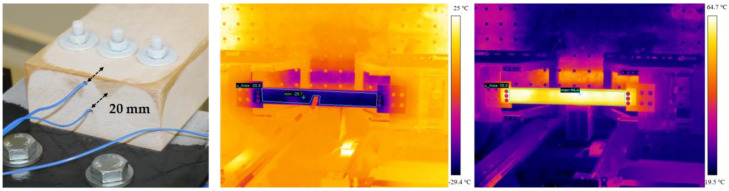
Position of the two temperature sensors in the end grain wood of the quasi-static tests (**a**) and exemplary images from the infrared camera for a −30 °C; (**b**) and a +90 °C; (**c**) sample.

The samples were conditioned for 24 h in a climate chamber or in a laboratory freezer, depending on the temperature stage. It was not possible to condition the whole testing facilities to the required temperature levels. Due to the fact that the thermal capacity of wood is rather low, the temperature was chosen 10 °C higher for the +60 °C and +90 °C samples and 10 °C lower for the −30 °C and 0 °C samples. In order to delay the unwanted cooling or heating and to minimize thermal radiation in the timespan between removal from the conditioning environment and the actual test, the samples were kept in a portable, custom-made climate chamber and wrapped in aluminum foil. To provide repeatable boundary conditions, care was taken to keep handling/process times similar. Prior to testing, the surface temperature of the specimens was measured with an infrared camera (see Figure 3). Due to the logical fact that the quasi-static test procedure was significantly longer than the dynamic one (roughly 15 min vs. only 20 ms), a thermometer PCE-T390 in combination with temperature sensors of type K (PCE Deutschland GmbH, Meschede, Germany) were used in order to document the temperature gradient over time. The temperature sensors were located in predrilled holes in the end grain wood on both sides of the beam (see Figure 3).

The density of each sample was determined according to ISO 13061-2-2014 10 01 [31] and based on the mass and geometry of small specimens (nominal dimensions 40 × 40 × 40 mm^3^), which were taken immediately after the test and close to the fractured zone (see Equation (1)).

(1) $$\rho_{u}=\;\frac{m_{u}}{V_{u}\;}\;\left[\frac{kg}{m^{3}}\right]$$

*ρ*_*u*_, density at a certain moisture content *u*; *m*_*u*_, mass of the moist wood; *V*_*u*_, volume of the moist wood;

These samples were also used for the evaluation of the moisture content according to ISO 554-1976 08 [32]. The moisture content was determined by measuring the mass of the sample immediately after mechanical testing (*m*_*u*_) as well as after the drying process in a kiln to a complete oven-dry state (m_*od*_). With the help of this data, the moisture content was calculated according to Equation (2).

(2) $$u=\;\frac{m_{u}-m_{od}}{m_{od}\;}\times 100\left[\%\right]$$

**Figure 4:**
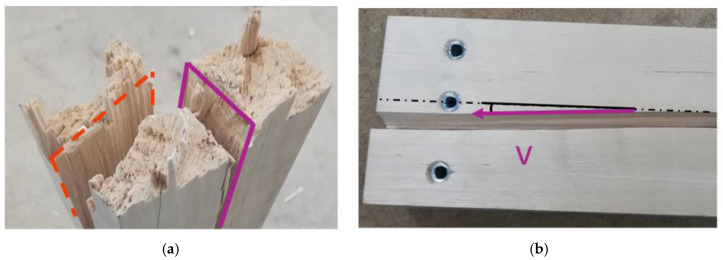
Split birch wood sample with primarily longitudinal-tangential (red, dashed line) and longitudinal-radial (magenta, solid line) surfaces (**a**); Split birch wood sample seen from the longitudinal-tangential plane with the highlighted fiber deviation of the longitudinal-radial plane (vector) relative to the beam axis (**b**).

*u*, moisture content; *m*_*od*_, mass of the oven dry wood;

To determine the fiber orientation in respect to a potential global fiber deviation, the samples were also split lengthwise in primarily tangential (red, dashed line) and radial directions (magenta, solid line) with the help of a chisel (see Figure 4).

The offset angle between fiber orientation and sample axis was evaluated in both planes with the help of a triangle ruler and converted into vectors. According to Götz and Kraft [33] in Equation (3), the spatial angle *θ* between fiber and sample axis was calculated section by section for each sample. For this purpose, the beam was divided lengthwise into four sections with a length of 200 mm each. The largest offset angle between the beam axis and the split surface was taken in order to have a representative value for each section.

(3) $$\theta =\;\arccos\left(\frac{u\;\times \;v}{\left|u\right|\;\times \;\left|v\right|}\right)\left[-\right]$$

*θ*, spatial angle between fiber and sample axis; *u*, vector in the first plane (red, dashed line); *v*, vector in the second plane (magenta, solid line).

### 2.3. Test Facility

**Figure 5:**
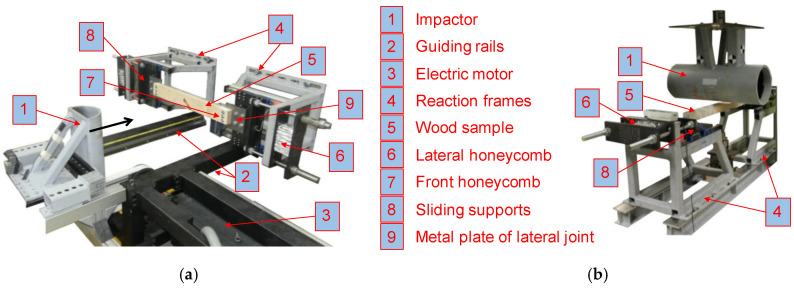
Test bench for the dynamic (**a**) and the quasi-static three-point bending test configuration (**b**).

The design of the test bench replicates the boundary conditions of a wood beam potentially used as a side-impact beam in the door of a passenger vehicle. In quasi-static bending tests, the impactor was displaced at 10 mm/min. In the dynamic tests, the initial velocity amounted for 8.89 m/s (mass = 91 kg). The dynamic speed of 8.89 m/s is related to the test specifications for a side-impact beam test according to FMVSS 214 [34] and therefore considered appropriate for the characterization of wood for crash-relevant purposes. As well as the testing velocity, the mass of the impactor also plays a crucial role for the impact energy in the dynamic tests. In the case of a side-impact, the pre-impact kinetic energy is converted to internal energy, friction, and post-impact kinetic energy over the whole vehicle. Only a small amount of the total energy is directly related to a side-impact beam. Therefore, a numerical study using a full vehicle model was conducted in order to obtain the maximum internal energy in a conventional metal side-impact beam. The full vehicle simulation indicated that the beam takes up 2500 J and the surrounding structure 1075 J. In the dynamic tests, the latter goes into the deformation of honeycomb elements (6) in Figure 5. Based on the initial velocity and the required impact energy (3575 J), the impactor mass was set to 91 kg.

The dynamic and the quasi-static test setup are shown in Figure 5. In the dynamic tests, the impactor (1) was driven through an electric motor (3) which accelerated a sled through a drive belt. According to FMVSS 214 [34], the diameter of the impactor was chosen to 254 mm (10 inches). The impactor (1) as well as the laterally sliding supports (8) on each side of the wood sample (5) were equipped with piezoresistive, uniaxial 1000 g ASC 62C1 accelerometers and a sampling frequency of 50 kHz (ASC GmbH, Pfaffenhofen, Germany). The acceleration signals were filtered by means of a CFC 180 Butterworth filter and multiplied with the impactor mass in order to obtain the force signal. Additionally, the whole testing process was filmed with two Macro VIS high speed cameras (one from the top and one from the side) at a frame rate of 1000 fps (Weinberger GmbH, Haan, Germany). The obtained images were also used for target tracking. As well as the distance measurement through target tracking, the impactor displacement was recorded with a LK-G507 laser at a sampling frequency of 50 kHz (Keyence Deutschland GmbH, Neu-Isenburg, Germany).

The quasi-static tests were conducted with a universal testing machine Zwick/Roell Z100 (Zwick GmbH & Co. KG, Ulm, Germany), equipped with a 100 kN load cell and the control software Zwick/Roell testXpert Ⅱ V3.5 (Zwick GmbH & Co. KG, Ulm, Germany). The displacement was measured with a conventional mechanical extensometer (MacroXtense, Zwick/Roell, Ulm, Germany). A displacement-controlled setting with a loading velocity of 10 mm/min was chosen for the quasi-static tests. However, there was no time limit considered for reaching the maximum force. The distance between the bearing points was 800 mm for the quasi-static as well as for the dynamic tests. The reaction frames (4) and the clamping conditions were identical to the dynamic tests. As well as the force-deformation characteristics, the bending strength was also evaluated. This was done with the help of the internal calculation routine of the control software Zwick/Roell testXpert Ⅱ V3.5 (Zwick GmbH & Co. KG, Ulm, Germany).

**Figure 6:**
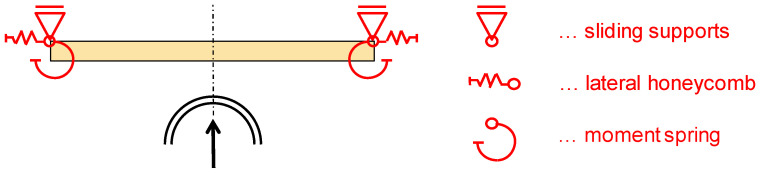
Static system of the wood specimen within the test bench.

The wood samples (5) were fixed on the laterally sliding supports (8) through metal plates (9). The metal plates of dimensions (165 × 160 × 4 mm^3^) were made from mild steel S 235. The laterally sliding supports (8) allowed for a translation orthogonal to the impactor displacement (see Figure 6). However, this degree of freedom is constrained through lateral honeycombs (6) with a batchwise calibrated crush force of 10.1 kN, each (aluminum honeycomb 3/8 3003, Cellbond, Huntingdon, UK). The crush force of 10.1 kN was obtained through numerical simulations with multiple full vehicle models and represents the stiffness and yielding force of the car door and the surrounding structure. The honeycombs (6) were pre-crushed in order to remove the initial force peak needed for initiation of buckling and to obtain an almost constant crush force plateau of 10.1 kN. Therefore, a linear relation between the deformation of the honeycombs and the absorbed energy could be assumed. The metal plates (9) acted as an elasto-plastic moment hinge designed to yield. The wood member was bolted to the metal plates (9) through three M6 8.8 bolts on each side. In order to increase the resistance of wood against an embedment failure, the screw holes were equipped with steel sleeves. The metal plates itself were fixed on the sliding supports with the help of four M12 8.8 screws. The stiffness and yield force of the lateral honeycombs (6) and the yield torque of metal plates (9) were designed in order to mirror closely the mounting situation in a real vehicle—again based on numerical simulations with full vehicle models. To finally stop the impactor in the dynamic tests, a honeycomb (7) was mounted to the crashblock.

**Figure 7:**
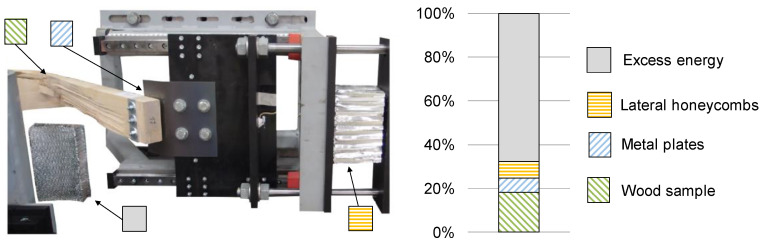
Schematic distribution between the energy absorbing elements in the test bench of the dynamic tests.

Within the dynamic tests, three main elements were responsible for absorbing the introduced energy (see Figure 7): firstly, energy is absorbed by the wood sample itself (light green, hatched diagonally downwards); secondly, energy is also absorbed through the deformation of the metal plates (light blue, hatched diagonally upwards). The deformation of the screws was not evaluated separately due to its small proportion. Thirdly, some of the initial kinetic energy is taken up by the calibrated aluminum honeycombs, positioned laterally (orange, hatched horizontally). Excess energy which could not be absorbed by the first three positions is taken by another calibrated aluminum honeycomb mounted on the crash-block in front (grey, hatched solid). The sum of these shares equals 100% and stands for the 3575 J of the initial kinetic energy.

The calculated energy absorption of the wood sample ends at maximum deformation which was defined as the deformation at the complete separation of the beam into two parts. This point of complete softening was determined with the help of the high-speed camera. The impact bending energy of the samples was calculated by dividing the internal energy of the wood sample by the cross-section (B = 90 mm × H = 43 mm) of the beam.

Due to the fact that the quasi-static tests were conducted displacement-controlled, the introduced energy varies with the maximum possible deformation of each specimen. The impactor was immediately stopped when complete softening or fracture over the whole cross-section was reached, i.e., no additional crash absorber (grey, hatched solid) was necessary. The calculated energy absorption of the wood sample ends at maximum deformation, i.e., at complete softening.

## 3. Results

### 3.1. Density, Fiber Deviation, and Climatic Conditions

The mean densities (ρ12, mean) relate to a reference moisture level of 12% and reach from 574 kg/m^3^ up to 649 kg/m^3^ (see Table 2). The reference densities were calculated according to Kollmann [15] and ISO 554–1976 08 [32]. The evaluation of the fiber orientation (θ) shows that about 85% of the samples had a fiber deviation less than 6°. Even the remaining 15% of the samples with slightly higher deviations (single values between 6° and 9° except for one sample with 15°) do not feature significantly lower force and energy-absorbing capabilities. The fiber orientation was only investigated in the samples of the dynamic tests.

An overview of the climatic boundary conditions which were achieved during the tests is shown in Table 2. Mean values of temperature and moisture content as well as the density and the fiber deviation are listed. Additionally, statistics of the coefficient of variation (CV), as a measure for the variation within each test configuration (*n* = 6), are included. In order to better illustrate the scattering of the temperature values, the standard deviation (σ) was used. In the case of the dynamic tests, average values from the surface temperature at test start determined by infrared camera were applied (test duration roughly 20 ms). For the quasi-static tests, which took about 15 min, the start temperature and the temperature gradient are given in the main statistics. Due to the high thermal insulation properties of wood, it can be assumed that during that duration, the core temperature remained close to the target temperature. Consequently, in the following, the temperature data always refers to the originally introduced nominal temperature levels. The moisture content (u) remained almost constant for the samples between −30 °C and +30 °C. When it comes to the upper temperature levels (+60 °C and +90 °C), moisture contents were significantly lower.

### 3.2. Force-Deformation Behavior

**Figure 8:**
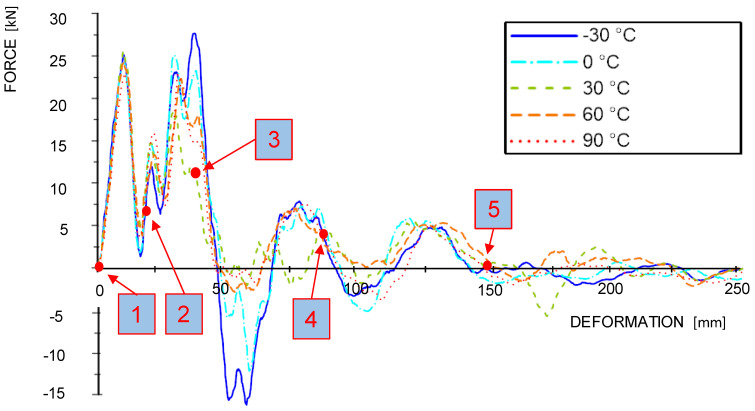
Average force-deformation curves of the dynamic tests.

**Figure 9:**
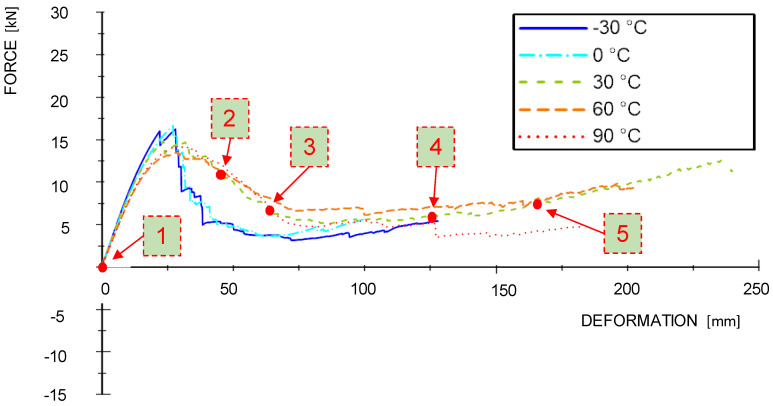
Average force-deformation curves of the quasi-static tests.

In order to compare the material reaction between the individual temperature levels, the results are plotted in force-deformation-diagrams (see Figure 8). These diagrams show the mean curves obtained from the dynamic and quasi-static tests which were calculated from the mean gradient of the single test curves and afterwards trimmed when reaching the mean value of the energy absorption. It can be observed that the stiffnesses and the achieved maximum forces in the dynamic tests are significantly higher than in the quasi-static tests. Additionally, the temperature level shows an influence on the material characteristics, namely on maximum force and the softening behavior. The maximum force in the dynamic tests ranged from 25 kN to 30 kN and in the quasi-static tests from 12.5 kN to 15 kN. Maximum forces decrease with increasing temperature (see Figure 8 and Figure 9). Obviously, there is also a positive correlation between temperature and ductility regarding to the rapidness of the force drop after reaching the maximum level.

There are negative force recordings in the dynamic tests from −30 °C and 0 °C samples starting approximately at 50 mm deformation. These negative forces are reasoned in the acceleration-based measurement: during the impact, elastic internal energy builds up in the test-setup (sled, linear bearings, guiding rails). When specimens fail rapidly, i.e., in a brittle manner, the internal (i.e., elastic) energy of the test setup is released suddenly, causing a “negative deceleration”. These overshoots hardly occur in the more ductile samples, i.e., at elevated temperatures between +30 °C and +90 °C. However, most of the samples show two smaller force-peaks at approximately 80 mm and 130 mm deformation prior to total softening at approximately 150 mm.

Effects from temperature were also observed in the quasi-static tests: there is a significant difference in the softening behavior between the mean-curves at lower (−30 °C and 0 °C) and the higher temperatures (+30 °C, +60 °C and +90 °C), i.e., below (water in frozen state) and above zero degree Celsius (water in liquid state). While there is a rather rapid softening in samples in the lower temperature levels, the behavior of samples at higher temperatures is in comparison rather ductile. The maximum average deformation prior to failure at the two lower temperature levels amounts for 100 mm and 130 mm, respectively, while gaining between 180 mm and 240 mm at the upper two temperature levels. The samples at +30 °C show the highest quasi-static deformation values.

In the quasi-static test curves, the force level is increasing again at roughly 100 mm. This is due to the partial lateral confinement provided through the honeycombs. As a result, tensile stresses are building up in the remaining effective cross-sections of the beams. The positions 1 to 5 in Figure 8 and Figure 9 are related to the failure sequences of an exemplary +30 °C sample, which are further described in the following Section 3.3.

**Figure 10:**
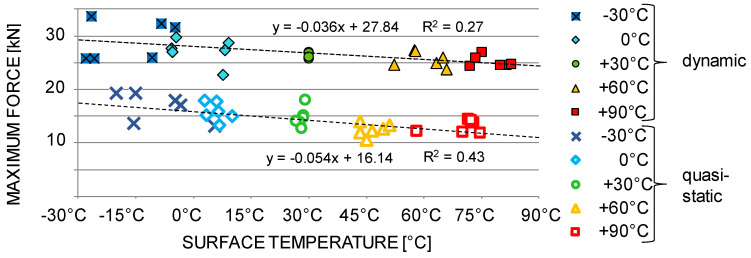
Scatter-plot of the dynamic and quasi-static maximum force over temperature.

Individual maximum force values in relation to the temperature level are shown in Figure 10. On average, the maximum forces in dynamic tests are twice as high as in quasi-static tests. However, the qualitative trend of the force-temperature relation is almost the same for the dynamic and the quasi-static case.

### 3.3. Failure Sequence and Fracture Characteristics

**Figure 11:**
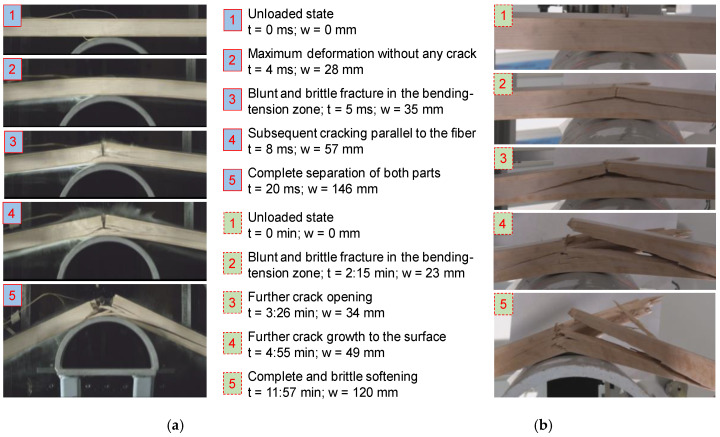
Exemplary loading and failure sequence in a dynamic (**a**) and a quasi-static sample (**b**) in series −30 °C.

In order to better understand the deformation and fracture behavior of the dynamic and the quasi-static tests, images showing the failure sequences of quasi-static and dynamic tests are compared against each other. Two temperature levels were exemplarily chosen, namely −30 °C and +30 °C, representative of a rather brittle and a rather ductile failure, respectively. The two temperature levels allow for an evaluation of the pure temperature effect, because the moisture content in the samples were fairly similar (9% to 10%). Figure 11 shows images taken at five different time steps with focus on the softening behavior in dynamic and quasi-static tests at −30 °C. A short description related to each image is added which contains the time (t) and the deformation (w) and allows relating between the loading and failure sequence and the force-deformation-diagrams as given in Figure 8 and Figure 9. Both the dynamic and the quasi-static tests show a blunt failure in longitudinal tension in the bending-tension zone opposite to the impactor followed by subsequent cracking parallel to the fiber direction towards the supports until final and complete softening occurs.

**Figure 12:**
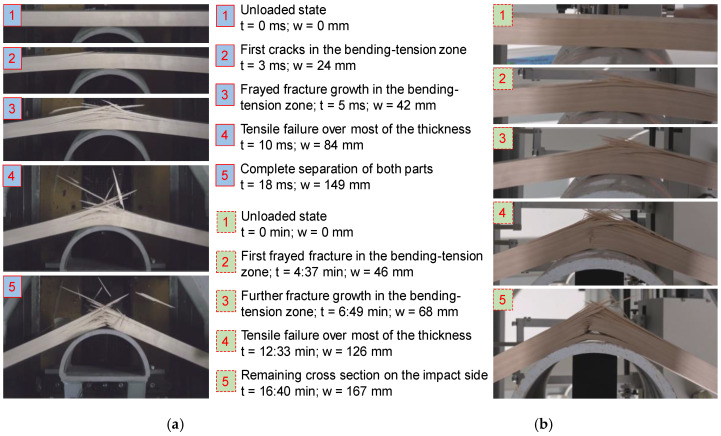
Exemplary loading and failure sequence in a dynamic (**a**) and a quasi-static sample (**b**) in series +30 °C.

Samples conditioned at +30 °C show a more ductile fracture behavior, as demonstrated in Figure 12, with strong defibration and pronounced fiber bridging on the tensile side. Similar to the samples in series −30 °C, the first cracks appear on the tension side in the bending-tension zone, opposite to the impactor (=far side). However, in this case, the tension failure is much more fragmented, and the crack pattern is more uniform. Especially in the quasi-static load case, crack-growth is rather slow and ductile, allowing for a load-transfer to the remaining effective cross-section on the front side of the impactor. The front side, which is the bending-compression zone, had been mainly stressed in compression parallel to the fiber direction, and in addition, in compression perpendicular to the fiber direction in the center part impacted by the impactor. At this stage, the majority of the cross-section has failed due to longitudinal tension. Since the samples are partially confined in their lateral displacement, the stress state changes from bending to almost pure tension, leading to crushing of the lateral honeycombs. As a result, the force in the quasi-static tests is slightly increasing (see Figure 9). In the dynamic case and contrary to the samples which were conditioned at −30 °C, samples conditioned at +30 °C show a pronounced splintering.

### 3.4. Energy-Absorbing Capabilities

**Figure 13:**
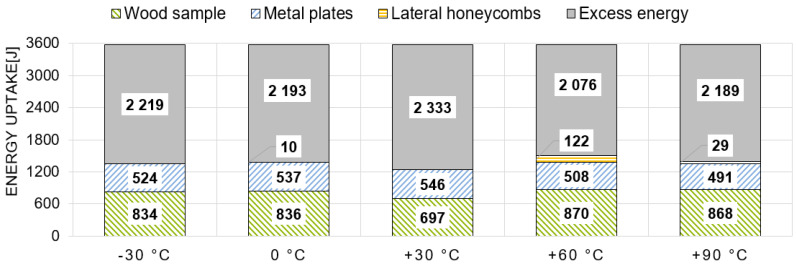
Energy distribution of the samples under dynamic loading in tested temperature series, restricted to internal energy.

Another important aspect of this study is the energy-absorbing capability. As already described in Section 2.3, there are three major energy absorbing elements: the wood sample itself, the metal plates and the lateral honeycombs. Excess energy is absorbed by the front honeycombs, which accounts for roughly 60% of the input energy. Figure 13 shows the mean values of the energies by temperature within the dynamic tests. On average, about 19% to 24% of the energy uptake is attributable to the wood sample itself (697 J to 870 J). The metal plates are accounting for roughly 15% of the energy absorbing capability, i.e., yielding of the joint. The share of energy absorption by the lateral honeycombs is by far the smallest contribution. Apart from the samples, which were conditioned at +60 °C, this amount is negligibly small or even zero. Consequently, as the partial lateral confinement was not effectively activated, the samples failed under almost pure bending.

**Figure 14:**
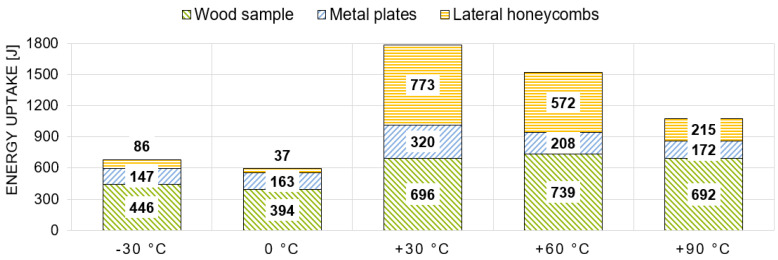
Energy distribution of the samples under quasi-static loading in tested temperature series, restricted to internal energy.

In the case of the quasi-static tests, the energy distribution is quite different (see Figure 14). First of all, there is a huge variation in the total absorbed energy regarding the temperature levels. This variation is way larger than in the dynamic tests and primarily effected by the different activation level of the honeycombs on the one hand, but also by the wood sample reaction on the other hand. While there is almost no activation at the lower temperatures (−30 °C and 0 °C), the energy absorption of the lateral honeycombs at the higher temperature levels, especially at +30 °C and +60 °C, is quite significant. The energy taken up by the wood samples in the lower temperature range (−30 °C and 0 °C) is on average half as much as in the dynamic configuration and thus similar to the ratio between the maximum forces. On the other hand, the share of energy absorption by the wood samples conditioned between +30 °C and +90 °C is only slightly lower (between 0% and 20%) than in the dynamic tests.

## 4. Discussion

### 4.1. Density, Fiber Deviation, and Climatic Conditions

The mean density of the samples at a reference moisture content of 12% (ρ12, mean) was between 574 kg/m^3^ and 649 kg/m^3^, which is a quite common value range when comparing the data with literature; e.g., Wagenführ [23] (ρ12 = 510 kg/m^3^ to 830 kg/m^3^) or Grabner [24] (ρ12, mean = 620 kg/m^3^). The variability of the density values within the individual temperature levels was between 3.9% and 10.9% (see Table 2). This can be considered a quite common density scattering of solid wood: e.g., Kretschmann [26] (CV [ρ12] = 10%) or Missanjo and Matsumura [35] (CV [ρ12] = 6.6%). In order to identify possible effects of density on the energy uptake, additional impact bending tests according to ISO 13061-10-2017 10 [36] were conducted. Overall, 28 solid birch wood samples (20 × 20 × 300 mm^3^) were tested at standardized climate (+20 °C and 65% relative humidity). The density was calculated according to Equation (1) and related to a reference moisture content of 12%.

**Figure 15:**
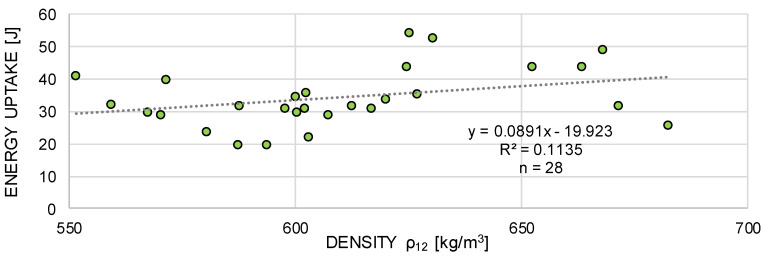
Correlation between energy absorption and density of the solid birch wood samples tested under standardized climate conditions.

**Figure 16:**
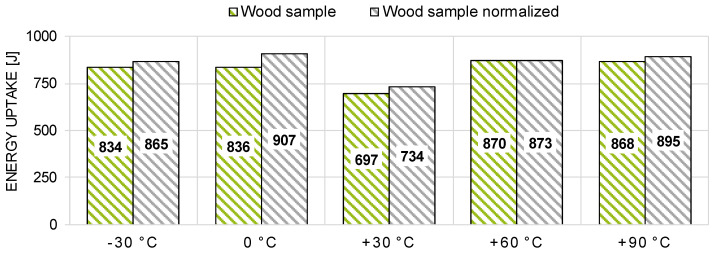
Comparison between the dynamically tested mean energy uptake of the wood samples against a normalized mean energy uptake.

The coefficient of determination between the density and the impact bending energy of the specimens was found to be low (linear regression model with a coefficient of determination R2 = 0.11; see Figure 15). The statistically weak correlation between the density and the energy uptake of wood is also reported in literature (see Burcar and Merhar [17], Wilson et al. [37], and Reiterer et al. [38]). Despite the low R^2^, the established linear regression function was used to normalize the energy uptake to a reference density (606 kg/m^3^, i.e., the mean density over all test series). A comparison between the dynamically tested energy uptake of the wood samples against a normalized energy uptake is shown in Figure 16.

The normalization has no major effect on the results and the conclusion. In order to statically confirm the negligible influence of the density variability and the deviation of the mean density values between the temperature levels, two hypothesis tests were carried out. The tests were conducted with a significance level of 5% and under the assumption that the density has an unknown variance but is approximately normally distributed. The mean density values of the individual temperature levels were compared pairwise within a test series (quasi-static or dynamic) and across the test series.

For the first hypothesis (no difference in density mean values), a Welch test (adapted T-test) was applied. Within each test-series, no hypothesis was rejected (10 out of 10 pairwise combinations). Across the test-series, the hypothesis was rejected in 4 out of 25 combinations).

For the second hypothesis (quotient between the variances of two samples equals unity), a F-test was applied. It showed that the hypothesis was never rejected in static tests, one time (out of ten) in the dynamic tests, and only two times (out of 25) across test conditions.

Consequently, it can be assumed that the influence of the density on the conclusions made regarding temperature is negligible and statistically insignificant. This is true for the position (mean values; Welch-T-test, 5%) as well as for the dispersion (variances; F-test, 5%).

About 85% of the obtained fiber deviations (θ) were lower than 6°, which, considering for example the Hankinson model (see Kim [39]) and the low number of replicates within each series, is too low in order to statistically identify a significant influence on the mechanical properties. According to Kollmann [15] the bending strength of ash wood at a fiber deviation of 6° drops by about 8.5%. Additionally, the few samples with slightly higher fiber deviations do not feature significantly lower mechanical properties. The moisture content was almost constant for the temperature between −30 °C and +30 °C, while it decreased significantly at the higher temperature levels (+60 °C and +90 °C). According to Kollmann [15], there is an exponential growth of the diffusion coefficient with increasing temperature and therefore the drying process goes much faster at elevated temperatures. This means that due to the drying out of the samples, it was not possible to study the pure temperature effect without changing the moisture content at these temperature levels.

However, there is also a certain variation in the obtained moisture content within the individual temperature stages. Due to the time span between mechanical testing and the moisture sample preparation as well as the weighing process, the moisture content could have changed to some extent. This could be one explanation why there is some variation in the moisture level of the samples. On the other hand, there is also some variation on the relative humidity during the conditioning of the samples which also effects the moisture content of the samples. This is due to the fact that it was not possible to regulate the humidity neither in the used climate chamber nor in the laboratory freezer.

Several climate-related effects have an influence on the material behavior: as already indicated, the temperature and moisture content of the wood samples, which are open to diffusion, cannot be decoupled from each other. In the case of the temperature distribution within the beams, there is a certain gradient between the inner core and the outer surface area. Therefore, the temperature in the tension and compression edge fibers was different from the primarily shear-stressed core area. This circumstance led to a rather complex interaction between different stress states and temperature areas over the cross-section. Due to the rather small number of samples, there is a statistically greater dispersion for the individual series. The deviation is particularly high for those temperature levels (−30 °C and + 90 °C), which show a greater difference to the actual temperature at the testing facility. This is due to the more pronounced temperature equalization at larger temperature gradients. Due to the fact that water, which is bonded in the cell walls, partially goes into the lumina and starts freezing between −2 °C and −6 °C, the situation for the 0 °C samples is especially complex (see Wimmer et al. [14]). It can be assumed that the outer areas of the samples conditioned at 0 °C were already defrosted while the core was still frozen.

### 4.2. Force-Deformation Behavior

Wimmer et al. [14] further mentions that frozen water supports the cell structure and therefore leads to an increase of maximum force and stiffness. Due to the fact that the −30 °C and 0 °C samples were preconditioned at 10 °C lower, it can be assumed that the enclosed water was already frozen at both temperature levels. This explains the higher maximum forces in the dynamic and quasi-static tests (see Figure 8 and Figure 9). Zhao et al. [40] has studied the flexural behavior of birch wood at varying temperature and moisture conditions under quasi-static loading. One of their major findings is that both the free and the hygroscopic frozen water are affecting the ductility ratio, which is a deformation relation between the first nonlinearity and a post peak force drop of 15%. The frozen water restricts the movement of the cellulose molecules, making the material apparently more brittle. The same phenomenon can be observed on the conducted dynamic and quasi-static tests. On the other hand, the material behavior, in terms of maximum force and ductility, remained almost unchanged in the temperature range between +30 °C and +90 °C. According to Gerhards [19], an increase in temperature leads to a decrease of the maximum force, while a decrease of the moisture content has the opposite effect. Solid wood at varying moisture content shows a glass transition temperature in the range of +60 °C to +115 °C (Zhou et al. [41]). However, according to Zhou et al. [41], the softening of hemicellulose (polyoses) and lignin already starts at temperatures between +30 °C and +70 °C. It seems that moisture content and temperature, which are countercurrent in the experiments, almost cancel out each other to a certain extent. The temperature effect also depends on the fiber direction. Gerhards [19] and Salmen [42] have shown that properties perpendicular to the fiber direction are more sensitive to temperature changes than the one parallel to the fiber. In terms of the average force level over the temperature series, the dynamic values are roughly twice as high as the quasi-static ones. Gilbertson and Bulleit [43] have conducted compression tests on maple and pine at high strain rates by using a Split Hopkinson pressure bar. The compressive strength, which was determined at strain rates between 69/s and 337/s, was roughly between 2 and 2.4 times higher than the values of the reference samples at quasi-static loading. Analyzing the dynamic force deformation characteristics, there are two major force peaks within the first 50 mm of deformation. According to Bröker and Salamon [44], who have instrumented a pendulum for testing wooden samples, the first force peak results from an acceleration shock (compare Figure 8). Due to the acceleration shock, the impactor temporally loses contact with the sample, which explains the sudden force drop. The force increases when the impactor makes contact again.

### 4.3. Failure Sequence and Fracture Characteristics

The failure sequences of the samples have shown that fracturing of the samples over all temperature series primarily occurs in the bending-tension zone. There were only rather small compression folds visible in the bending-compression zone, although partial softening in the bending-compression zone leads to a delay of the fracture in the bending-tension zone. According to Wimmer et al. [14], the failure type in a bending sample highly depends on the moisture content and the temperature. Gerhards [19] also pointed out that the compression strength of wood parallel to the grain is much more increased by a reduction of the moisture content than the tensile strength parallel to the grain. In the case of the observed samples in the present study, the moisture content was rather low (between 3.6% and 10.1%). This explains why the failure was primarily visible in the bending-tension zone rather than in the compression zone.

Wood fibers boast a theoretical tensile strength of 400 N/mm^2^. In practice, this strength cannot be achieved in a wooden member during tensile tests, because under the influence of existing shear stresses and the low transverse strength of the wood, a lateral sliding of the fibers occurs, with the individual fiber bundles tearing at their weakest points. Characteristic for this phenomenon is also the long-fibered, splintering fracture structure during the tensile test parallel to the fiber (see Kollmann [15]). It was shown that by increasing the transverse strength, e.g., through glue impregnation of veneers, that the fracture pattern was becoming short-fibered and that tensile strength parallel to the grain can be increased (see Kraemer [45]).

On a cellular scale, fracture paths in wood can be distinguished in cell fracture and cell separation, also sometimes referred to as intra- and inter-cellular fracture, respectively (see Boatright and Garrett [46]; Ashby et al. [47]; DeBaise [48]). Intracell failure can be further separated into transwall (separation across the entire cell wall) and intrawall (separation within secondary wall) failure (see Koran [49]). Cell fracture occurs mainly in low-density woods or in earlywood of higher density woods. In higher density woods, with minor differences between early and late wood, such as birch, cell separation through the middle lamella and primary cell wall is more common (see Conrad et al. [50]). As a result, low-density woods (intracellular failure) will typically show Radial-Tangential (RT), Radial-Longitudinal (RL), Longitudinal-Radial (LR), and Longitudinal-Tangential (LT) crack direction. Intercellular failure in higher density is typically associated to TR direction (see Ashby et al. [47]). However, in higher density hardwoods, still intracellular failure, namely a transwall failure, can be observed when loaded parallel to the grain. In this case, the failure follows the S2 fibrillar angle in a spiraling manner, likely due to slippage between microfibrils (Cote and Hanna [51]).

On a macroscopic scale, wood shows a toughness against fracture: in Mode I, the opening mode, cracks will propagate parallel to the grain (RL, TL, RT, TR) and far less likely perpendicular to the grain (LR and LT), as the fracture toughness perpendicular to the grain is one order of magnitude greater than parallel to the grain. This can also be explained through the higher fracture toughness of cell fracture as opposed to cell separation (see Conrad et al. [50]). As a result, a crack (or notch) in the LR or LT direction will deviate to the weaker planes, i.e., the crack will follow the path of minimum crack resistance and propagate under mixed mode conditions. On a macroscopic scale, it is virtually impossible to propagate a Mode I fracture perpendicular to the grain without inducing Mode II parallel to the grain (see Conrad et al. [50]). Due to the “duration-of-load effect”, which describes the time for cracks to reach a critical size, the fracture toughness increases with strain rate (see Blicblau and Cook [52]; Johnson [53]; Nadeau et al. [54]; Schniewind and Centeno [55]). Due to viscoelastic effects (see Mindess [56]), fracture toughness also increases with moisture content from oven-dry to *u* = 6–8% moisture content. After that, Mode I fracture toughness is constant or decreases (see Conrad et al. [50])—likely due to water ingress in micro fibrils, decreasing crystallinity. In wedge-splitting tests, a decreasing fracture toughness was determined for hardwoods, like beech in the RL (900 to 650 kN m-3/2) and TR system (550 to 500 kN m-3/2). Same applies to the specific fracture energy (600 to 500 N/m) in the RL system (see Vasic and Stanzl-Teschegg, [57]). Mode I fracture toughness also increases linearly or slightly exponentially with density perpendicular and parallel to the grain (see Ashby et al. [47]; Leicester [58]). Since the fracture toughness in opening mode perpendicular to the grain is very high and transverse tension in wood construction should be mitigated anyways, the Mode II, the in-plane shearing mode, contributes considerably to the failure of wooden structures. The Mode II fracture toughness can be determined for the TL and RL direction—but difficult for the other directions (see Barrett and Foschi [59]). Generally, though, the Mode II fracture toughness values are consistently higher that the Mode I values in the corresponding direction. Similar to Mode I, an almost linear relation to density can be established, however, the coefficient is smaller (i.e., the increase per density gain) (see Leicester [58]). DeBaise et al. [48] showed that the crack surface is getting rougher with higher crack propagation rates in coniferous woods.

To summarize, the fracture observed in the specimens are caused by natural weak planes along which a crack propagates. Variations in these fracture patterns are likely influenced by strain-rate and moisture dependency of fracture toughness along various fracture directions. One explanation might be an increase in transverse strength (similar as observed in glue-impregnated veneers), leading to shorter fracture fibers and less splintering.

### 4.4. Energy-Absorbing Capabilities

While the dynamic response on the energy absorption of the wood samples remained almost constant over all investigated temperature levels, in the quasi-static tests, there is a pronounced difference between the lower (−30 °C and 0 °C) and the upper (+30 °C, +60 °C, and +90 °C) temperature levels. Kollmann [15] has investigated similar effects on the impact bending energy of pine wood at temperatures −30 °C, 0 °C, and +20 °C. In his pine wood samples, which had an average density of 520 kg/m^3^, the impact bending energy values remained almost identical at all temperature levels.

Generally, the activation of the lateral honeycombs is a good indicator for the toughness of the material. Additionally, the loading velocity seems to play a role in the load transformation and therefore in the activation of the lateral honeycombs. In dynamic testing, the amount of absorbed energy was almost constant throughout all temperature ranges. This was not the case in quasi-static testing, where activation of lateral honeycombs occurred at upper temperature levels. At lower temperatures, the failure of the quasi-statically tested samples was too brittle in order to achieve a pronounced load distribution within the wood samples and a significant activation of the honeycombs. On the other hand, at elevated temperatures, there were mechanisms of load redistributions to the remaining intact cross-sections as well as to the honeycombs. The deformation of the honeycomb structures correlates quite well with the deformation of the samples in the quasi-static tests (compare Figure 9 and Figure 14).

### 4.5. Comparison of Bending Strength and Impact Bending Energy Values

**Figure 17:**
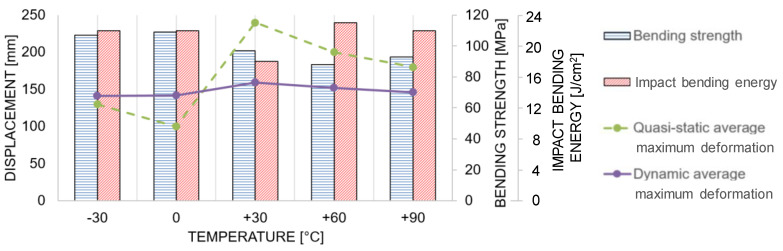
Mean values of bending strength and impact bending energy as well as the average maximum deformation of the quasi-static and dynamic tests over temperature.

The mean bending strength and the impact bending energies for the individual temperature stages are shown in Figure 17. Furthermore, also the average maximum deformation of both the quasi-static and the dynamic tests are plotted.

Given the reference climate according to ISO 554-1976 08 [32] (+20 °C and 65% relative humidity), values for quasi-static as well as impact bending energy of small clear wood specimens made of birch can be found in, e.g., Sell [25], Wagenführ [23], Grabner [24], and Kretschmann [26] (see Table 3 as a brief summary).

The values provided in Table 3 show a large variation for quasi-static bending strength (76 MPa to 155 MPa, which is approximately 200%) and even more so for the impact bending energy (4 J/cm^2^ to 17.5 J/cm^2^, which is approximately 440%). As some kind of average values, the quasi-static bending strength results in 118 MPa and the impact bending energy in 9.0 J/cm^2^.

The obtained values from the component size birch wood beams should give an idea for the order of magnitude but cannot be directly compared to the clear wood values from literature shown in Table 3. This is due to the fact that the sample geometry and scale as well as the degrees of freedom are different to a standardized clear wood characterization test, e.g., according to ISO 13061-3-2014 12 01 [60]. However, the bending strength value at +30 °C, which is closest to the standardized climate from literature (+20 °C and 65% relative humidity), is 18% lower than the mean value calculated from the literature (97 MPa vs. 118 MPa). In case of the impact bending energy, however, the obtained values as well as the impact velocity are almost twice as high as in a standardized clear wood test, according to ISO 13061-10-2017 10 [36] (18 J/cm^2^ vs. 9.0 J/cm^2^ and 32 km/h vs. 17 km/h).

## 5. Conclusions

The study has shown that the maximum forces of the wood samples in the dynamic tests are on average twice as high as in the quasi-static tests. Between −30 °C and +30 °C, the maximum forces (i.e., bending strength) decreased with increasing temperature but stayed almost constant in the temperature range from +30 °C to +90 °C. Although the boundary conditions of the component size specimens were not the same as on standardized clear wood tests, the mean quasi-static bending strength at +30 °C is comparable to the mean quasi-static bending strength value calculated from the literature (97 MPa vs. 118 MPa). In the case of the impact bending energy, the mean value from the component size tests is twice as high as the mean value calculated from the literature (18 J/cm^2^ vs. 9.0 J/cm^2^). In the dynamic tests and in terms of the deformation at failure, the value was almost independent of the temperature levels and approximately 150 mm. Within the quasi-static tests, the mean value of the deformation at complete softening ranged from 100 mm at 0 °C, over 130 mm at −30 °C, 180 mm at +90 °C, and 200 mm at 60 °C up to 240 mm at +30 °C. The accumulated energy uptake of the wood samples in the dynamic tests remained almost constant over the whole range of investigated temperatures with some tendency to be slightly higher at elevated temperatures. In the case of the quasi-static tests, the energy uptake at lower temperatures (−30 °C and 0 °C) was significantly smaller than at higher temperature levels (+30 °C to +90 °C). This was due to the more brittle failure mode at lower temperatures. The indicated temperatures always refer to the target temperatures, which are slightly different to the actual obtained values (compare Table 2). Furthermore, the temperature is not constant over the cross-section, which means that inner and outer beam zones are differently affected. A high correlation between the deformation of the sample and the activation of the honeycombs was investigated. Furthermore, the failure sequences and fracture patterns were analyzed on different temperature levels and loading velocities. In the case of the lowest temperature level (−30 °C), a blunt fracture pattern was observed in the dynamic as well as in the quasi-static tests. In contrast, the fracture pattern at +30 °C was finely frayed, apparently more uniform, and accompanied by load redistributions especially in the quasi-static case.

## Figures and Tables

**Table 1:** Matrix of the conducted experiments at different temperatures ranging from nominally −30 °C to +90 °C for dynamic and quasi-static bending tests.

Temperature	−30 °C	0 °C	+30 °C	+60 °C	+90 °C
Dynamic	*n* = 6	*n* = 6	*n* = 6	*n* = 6	*n* = 6
Quasi-static	*n* = 6	*n* = 6	*n* = 6	*n* = 6	*n* = 6

**Table 2:** Main statistics of the temperature, moisture content, and density values.

**Temperature-Level**	***n* (-)**	**T_mean_ (°C)**	**σ_T_ (°C)**	**u_mean_ (%)**	**CV (u) (%)**	**ρ_12,mean_ (kg/m^3^)**	**CV (ρ_12_) (%)**	**θ_mean_** **[°]**	**CV (_θ_) (%)**
Dynamic									
−30 °C	6	−17	10.4	9.4	17.0	592	9.0	2.6	60.2
0 °C	6	+2	9.1	9.3	15.1	574	4.9	5.1	50.9
+30 °C	6	+30	-	9.5	13.7	586	3.9	3.6	50.8
+60 °C	6	+60	5.4	6.5	41.5	605	10.7	5.1	104.1
+90 °C	6	+77	4.6	3.6	19.4	594	9.8	5.9	35.8
	***n* (-)**	**T_start_ (°C)**	**σ_T_ (°C)**	**ΔT (°C)**	**σ_Δ__T_ (°C)**	**u_mean_ (%)**	**CV (u) (%)**	**ρ_12,mean_ (kg/m^3^)**	**CV (ρ_12_) (%)**
Quasi-static									
−30 °C	6	−27	7.6	+25.1	7.5	10.1	7.9	599	10.9
0 °C	6	−1	1.1	+9.9	1.8	9.8	13.3	625	5.0
+30 °C	6	+32	1.7	−5.4	1.1	9.3	8.6	649	9.9
+60 °C	6	+61	1.0	−23.3	2.3	8.1	4.9	600	5.0
+90 °C	6	+89	4.8	−32.6	6.3	5.2	13.5	637	7.8

Temperature level, target core temperature; T_mean_, achieved mean surface temperature; u_mean_, mean moisture content; ρ_12,mean_, mean density; θ_mean_, mean fiber deviation; σ, standard deviation; CV, coefficient of variation; T_start_, achieved start core temperature; ΔT, temperature gradient over the testing duration.

**Table 3:** Average ranges of quasi-static and impact bending energy values for small clear wood specimens of birch.

Author	Sell [25]	Wagenführ [23]	Grabner [24]	Kretschmann [26]
Quasi-static bending strength (MPa)	120 to 144 *	76 to 155 **	112 ***	114 ****
Impact bending energy (J/cm^2^)	7.5 to 10 *	4.5 to 13 **	4 ***	14.1–17.5 ****

* *Betula verrucosa*; ** *Betula pendula* Roth.; *** *Betula* spp.; **** *Betula alleghaniensis*.

## References

[1] Müller C. (2015). Untersuchung von Holzwerkstoffen Unter Schlagbelastung zur Beurteilung der Werkstoffeignung für den Maschinenbau. Ph.D. Thesis.

[2] Müller U., Jost T., Kurzböck C., Stadlmann A., Kirschbichler S., Baumann G., Feist F. (2018). Crash Simulation of wood and composite for future automotive engineering. Wood Mater. Sci. Eng..

[3] (2014). ÖNORM EN 1991-1-7—2014-09: Eurocode 1: Actions on Structures—Part 1–7: General Actions–Accidental Actions (Consolidated Version).

[4] (2012). ÖNORM EN 1991-2—2012-03: Eurocode 1: Actions on Structures—Part 2: Traffic Loads on Bridges (Consolidated Version).

[5] (2013). ÖNORM EN 1991-3—2013-12: Eurocode 1: Actions on Structures—Part 3: Actions Induced by Cranes and Machinery (Consolidated Version).

[6] Goubel C., Massenzio M., Ronel S. Wood-steel structure for vehicle restraint systems. Proceedings of the 8th European LS-DYNA Users Conference in Strasbourg.

[7] Murray Y.D. (2007). Manual for LS-Dyna Wood Material Model 143.

[8] Shenoy M.M., Smith L.V., Axtell J.T. (2001). Performance assessment of wood, metal and composite baseball bats. Compos. Struct..

[9] O’Brien M. (2003). Finite Element Analysis of Wood and Composite Structured Hockey Sticks. University of Massachusetts at Amherst, Professor Ian Grosse, 605 Finite Element Analysis, ASME. http://www-unix.ecs.umass.edu/mie/labs/mda/fea/fealib/obrien/obrienReport.pdf.

[10] Ruggiero E., Sherwood J., Drane P., Kretschmann D. (2012). An investigation of bat durability by wood species. 9th Conference of the International Sports Engineering Association. Procedia Eng..

[11] Drane P., Sherwood J., Colosimo R., Kretschmann D. (2012). A study of wood baseball bat breakage, 9th Conference of the International Sports Engineering Association. Procedia Eng..

[12] Manin L., Poggi M., Havard N. (2012). Vibrations of table tennis racket composite wood blades: modelling and experiments, 9th Conference of the International Sports Engineering Association. Procedia Eng..

[13] Ruggiero E., Sherwood J., Drane P., Duffy M., Kretschmann D. (2014). Finite element modeling of wood bat profiles for durability, The 2014 conference of the International Sports Engineering Association. Procedia Eng..

[14] Wimmer R., Felber G., Teischinger A. (2011). Biegemechanische Eigenschaften von Fichte in Abhängigkeit von Feuchtigkeit und Temperatur. Holztechnologie.

[15] Kollmann F. (1982). Technologie des Holzes und der Holzwerkstoffe.

[16] Kretschmann D.E., Green D.W. (1996). Modeling moisture content-mechanical property relationships for clear southern pine. Wood Fiber Sci..

[17] Bucar D.G., Merhar M. (2015). Impact and dynamic bending strength determination of Norway Spruce by impact pendulum deceleration. BioResources.

[18] Tukiainen P., Hughes M. (2015). The effect of temperature and moisture content on the fracture behavior of spruce and birch. Holzforschung.

[19] Gerhards C.C. (1982). Effect of moisture content and temperature on the mechanical properties of wood: an analysis of immediate effects. Wood Fiber.

[20] Heräjärvi H. (2004). Static bending properties of Finnish brich wood. Wood Sci. Technol..

[21] Falconer J., Rivas B. (2013). De Havilland Mosquito Owners’Workshop Manual.

[22] (2019). ISO 3129–2019 11. Wood—Sampling Methods and General Requirements for Physical and Mechanical Testing of Small Clear Wood Specimens.

[23] Wagenführ R. (2006). Holzatlas.

[24] Grabner M. (2017). Werkholz: Eigenschaften und Historische Nutzung 60 Mitteleuropäischer Baum- und Straucharten.

[25] Sell J. (1997). Eigenschaften und Kenngrössen von Holzarten.

[26] Kretschmann D.E. (1999). Wood Handbook: Mechanical Properties of Wood.

[27] Shaharuzaman M.A., Sapuan S.M., Mansor M.R., Zuhri M.Y.M. (2018). Passenger car’s side door impact beam: A review. J. Eng. Technol..

[28] Baumann G., Stadlmann A., Kurzböck C., Feist F. (2019). Crashsichere Holzverbundwerkstoffe in Leichtbaukarosserien der Zukunft. Automob. Z. ATZ.

[29] Simlinger T. (2020). Experimentelle und Numerische Untersuchungen von Seitenaufprallträgern in Holzhybridbauweise. Master′s Thesis.

[30] Klee J. (2019). Experimentelle Untersuchung Eines Seitenaufprallträgers in Holzhybridbauweise. Bachelor Thesis.

[31] (2014). ISO 13061–2–2014 10 01. Physical and Mechanical Properties of Wood—Test Methods for Small Clear Wood Specimens—Part 2: Determination of Density for Physical and Mechanical Tests.

[32] (1976). ISO 554–1976 08. Standard Atmospheres for Conditioning and/or Testing–Specifications.

[33] Götz S., Kraft J. (2017). Mathematische Formelsammlung.

[34] FMVSS 214 (1992). Laboratory Test Procedure for FMVSS 214.

[35] Missanjo E., Matsumura J. (2016). Wood Density and mechanical properties of Pinus kesiya Royle ex Gordon in Malawi. Forests.

[36] ISO 13061–10–2017 10 (2017). Physical and Mechanical Properties of Wood—Test Methods for Small Clear Wood Specimens—Part 10: Determination of Impact Bending Strength.

[37] Wilson E., Mohammadi M.S., Nairn J.A. (2013). Crack propagation fracture toughness of several wood species. Adv. Civil Eng. Mater..

[38] Reiterer A., Sinn G., Stanzl-Tschegg S.E. (2002). Fracture characteristics of different wood species under mode I loading perpendicular to the grain. Mater. Sci. Eng..

[39] Kim K.Y. (1986). A note on the Hankinson formula. Wood Fiber Sci..

[40] Zhao L., Jiang J., Lu J., Zhan T. (2015). Flexural property of wood in low temperature environment. Cold Reg. Sci. Technol..

[41] Zhou J., Hu C., Hu S., Yun H., Jiang G., Zhang S. (2012). Effects of temperature on the bending performance of wood-based panels. BioResources.

[42] Salmen N.L., Fellers C. (1982). The Fundamentals of Energy Consumption during Viscoelastic and Plastic Deformation of Wood.

[43] Gilbertson C.G., Bulleit W.M. (2013). Load duration effects in wood at high strain rates. Am. Soc. Civil Eng..

[44] Bröker F.W., Salamon S. (1989). Instrumentierung eines Pendelschlagwerkes für die Holzprüfung. Holz als Roh-und Werkst..

[45] Kraemer O.J. (1934). Aufbau und Verleimung von Flugzeugsperrholz, Luftfahrtforschung.

[46] Boatright S.W.J., Garrett G. (1983). The effect of microstructure and stress state on the fracture behaviour of wood. J. Mat. Sci..

[47] Ashby M.F., Easterling K.E., Harryson R., Maiti K. (1985). The fracture and toughness of woods. Proc. R. Soc. London A.

[48] DeBaise G.R. (1972). Morphology of wood shear fracture. J. Mater..

[49] Koran Z. (1967). Electron microscopy of radial traceid of black spruce separated by tensile failure at various temperatures. Tappi.

[50] Conrad M.P.C., Smith G.D., Fernhund G. (2003). Fracture of Solid Wood: A Review of Structure and Properties at Different Length Scales. Wood Fiber Sci..

[51] Côté A., Hanna R.B. (1983). Ultrasound characteristics of wood fracture surfaces. Wood Fiber Sci..

[52] Blicblau A.S., Cook D.J. (1986). Aspects of wood fracture toughness at various testing speeds. Civil Eng. Trans..

[53] Johnson J.A. (1973). Crack initiation in wood plates. Wood Sci..

[54] Nadeau J.S., Bennett R., Fuller E.R. (1982). An explanation for the rate-of-loading and the duration-of-load effects in wood in terms of fracture mechanics. J. Mater. Sci..

[55] Schniewind A.P., Centeno J.C. (1973). Fracture toughness and duration of load factor I. Six principal systems of crackpropagation and the duration factor for cracks propa-gating parallel to grain. Wood Fiber.

[56] Mindness S. (1977). The fracture of wood in tension parallel to the grain. Can. J. Civil Eng..

[57] Vasic S., Stanzl-Tschegg S. (2007). Experimental and numerical investigation of wood fracture mechanisms at different humidity levels. Holzforschung.

[58] Leicester R.H. Application of Linear Fracture Mechanics to Timber Engineering. Proceedings of the Structural Integrity and Fractural International Conference (SIF’04).

[59] Barrett J.D., Foschi R.O. (1977). Mode II stress-intensity factors for cracked wood beams. Eng. Fract. Mech..

[60] (2014). ISO 13061–3–2014 12 01. Physical and Mechanical Properties of Wood—Test Methods for Small clear Wood Specimens—Part 3: Determination of Ultimate Strength in Static Bending.

